# Influence of Evidence-Based Nursing on Psychological Status, Neurological Function, and Life Quality of Patients with Acute Poststroke Depression

**DOI:** 10.1155/2022/7099908

**Published:** 2022-09-16

**Authors:** Yan Song, Fei Wang, Yakun Yang, Xing Liu, Chenghong Zhu

**Affiliations:** Department of Neurosurgery, The Characteristic Medical Center of PLA Rocket Force, Beijing 100088, China

## Abstract

**Objective:**

This research sets out to elucidate the influence of evidence-based nursing (EBN) on psychological status (PSY), neurological function, and quality of life (QoL) of patients with acute poststroke depression (PSD).

**Methods:**

One hundred and fifty stroke patients who received treatment in the Characteristic Medical Center of PLA Rocket Force between December 2019 and December 2021 were enrolled, including 100 cases (Group A) treated with comprehensive EBN and 50 patients (Group B) with routine nursing. Anxiety and depression (Self-Rating Anxiety Scale [SAS] and Self-Rating Depression Scale [SDS] scores), neurological function (National Institutes of Health Stroke Scale [NIHSS] and Scandinavian Stroke Scale [SSS] scores), QoL (Generic Quality Of Life Inventory-74 [GQOLI-74] score), and complication rate of both groups were evaluated, as well as total effective rate and nursing satisfaction.

**Results:**

Group A outperformed Group B with lower scores of NIHSS, SSS, SAS, and SDS and higher GOOLI-74 scores. Besides, lower complication rate and higher total effective rate and nursing satisfaction were determined in Group A.

**Conclusions:**

EBN can better improve the PSY of patients with acute PSD, restore their neurological function, and effectively improve their QoL.

## 1. Introduction

Stroke is a highly fatal disease that accounts for almost 5% of all disabling diseases and 10% of global deaths [1, 2]. Of these strokes, 85% are ischemic strokes [3]. Stroke is caused by blockage of intracranial blood vessels due to atherosclerosis. In most cases, these infarcts will block the blood flowing to a part of the brain, resulting in ischemic necrosis of the brain tissue supplied by these blood vessels, consequently inducing infarction [4]. Suffering from such a disease will not only adversely affect patients' activities of daily living to a great extent but also compromise their normal limb motor function and even cause consciousness in severe cases that may eventually lead to disability or death [5, 6]. Therefore, clinically, the treatment of stroke has always been an important research direction. For instance, patients receiving endovascular therapy have better physical recovery than those who receive standard drug treatment [7]. But in addition to treatment, the choice of nursing models is also critical to the posttreatment management of the disease, which is closely associated with the recovery of patients' various functions. This study mainly explores the influence of nursing mode on the psychological status (PSY) and neurological function in patients with acute poststroke depression (PSD), which is of great significance for improving patients' clinical symptoms, quality of life (QoL), and optimizing the management mode of such patients.

Evidence-based nursing (EBN) is a care method able to improve the treatment effect while reducing the medical cost [8]. This kind of nursing, developed based on real and reliable scientific evidence, is transforming the traditional narrow empirical nursing mode into a new nursing concept [9]. This nursing approach scientifically integrates the patient's will and nursing staff' clinical experience with relevant theories, which is also the basis for decision-making in the nursing process [10]. Nowadays, EBN has become an essential nursing means in medical institutions. Due to its high requirements for medical personnel, the demand for relevant skills training is also increasing [11–13]. Currently, EBN is extensively applied in the treatment of breast cancer and other gynecological cancers [14, 15], but there is scant research on its application in cardiocerebrovascular diseases. Consequently, this study studies its influence on stroke through PSY, neurological function, and QoL.

## 2. Methods

### 2.1. General Information

The subjects of this retrospective study were 150 stroke patients hospitalized in the Characteristic Medical Center of the PLA Rocket Force between December 2019 and December 2021. According to the care model they received, they were assigned to Group A (EBN) with 100 cases and Group B (routine care) with 50 cases. The two cohorts differed insignificantly in sex, age, and average monthly income (*P* > 0.05), with comparability. Group A was nursed by EBN methods, and Group B was nursed by routine nursing methods for patients with stroke. *Inclusion criteria are as follows*: diagnosis of stroke by cranial computed tomography (CT) or magnetic resonance imaging (MRI); no mental disorders; ability to express physical discomfort accurately; stable disease in recent one month, with no new episodes; and high compliance throughout the nursing procedures. *Exclusion criteria are as follows*: multiple basic diseases, engagement in other clinical trials, noncompliance with nursing or treatment, other brain diseases, and patients during lactation or pregnancy. The patients' family members were informed and signed the consent form. The hospital ethics committee ratified this study without reservations.

### 2.2. Methods

Both groups received thrombolytic therapy for acute stroke, during which Group B was given routine care. The medical staff provided rehabilitation guidance for patients, monitored patients' changes of vital signs, and improved their confidence in treatment, while Group A received EBN on the basis of Group B. First, an EBN team was set up. All team members received training on EBN knowledge to master effective methods of EBN, such as how to carry out effective rehabilitation training to help stroke patients improve their motor function and self-care ability, how to reduce or eliminate mental disorders of different degrees in stroke patients, and how to reduce various complications. In addition, the medical staff of the EBN team consulted relevant data for evidence-based support and used the network to search related literature to evaluate the authenticity, reliability, and clinical practicability of the evidence, so as to determine the conclusion. After that, the best nursing scheme was developed to carry out rehabilitation nursing for patients and guide them to carry out rehabilitation training. For paralyzed patients, special attention was paid, and corresponding measures were taken to ensure patient safety. For example, bedside rail restraint was used to prevent them from falling, and frostbite or burns were prevented. Besides, stroke patients were encouraged and assisted to carry out early activities as early as possible. Those who could get out of bed were helped to ambulate, sit down, and stand, so as to improve tolerance of the upright position. For patients without active participation ability, the nursing staff instruct them good limb position placement, shortening the time of lying on the affected side, performing massage and passive movement, and carrying out turning over training and bridge training, so as to promote autonomic nerve recovery and prevent contractures and joint deformities of the affected limbs. Language training was provided for patients with language impairment: First, the degree of aphasia was determined, and attention was paid to the most effective communication method retained by the patient. The language exercise was given step by step and followed the principle of from simple to difficult and from shallow to deep, with the content being continuously increased or updated according to the patient's acceptance ability, so as to avoid complications and diversification. Besides, appropriate physical, occupational, and music therapies were used to promote the treatment and rehabilitation of stroke patients. Psychological care for patients: early psychological intervention was also given to patients. Nurses actively communicated with patients and cared for them in a timely manner. In addition, health education was conducted, and publicity materials about stroke were distributed to patients to enhance their disease awareness. Meanwhile, the nursing staff understood the causes of the patients' bad psychology and took corresponding measures to solve the problem. Stroke-related knowledge was explained to patients, and the early rehabilitation training plan combined with the actual situation of each patient was worked out to solve the specific problems. Furthermore, patients were given material and spiritual support and encouragement. By enumerating some cases with good functional recovery and introducing their rehabilitation experience, the nursing staff helped patients establish the confidence to overcome the disease and the determination to adhere to treatment. Moreover, nurses communicated with the patient's families to provide all-round family support and social support to prevent patients from feeling loneliness. And patients' various complications were treated to reduce disease occurrence.

### 2.3. Detection Indicators

#### 2.3.1. Neurological Function

Patients' neurological function before and one month after nursing was compared. The assessment of patients' neurological recovery used the National Institutes of Health Stroke Scale (NIHSS; score range: 0-42 points) [16, 17] and the Scandinavian Stroke Scale (SSS; score range: 0-58 points) [18], the scores of which were inversely proportional to the patient's neurological recovery.

#### 2.3.2. Mental Health (MH)

Before and 1 month after nursing care, patients' MH was assessed using the Self-Rating Anxiety/Depression Scale (SAS/SDS) [19, 20] (20 items, 0-100 points), with higher scores representing worse MH.

#### 2.3.3. QoL

QoL after nursing was evaluated and compared based on the Generic Quality Of Life Inventory-74 (GQOLI-74) [21]. With a score ranging from 0 to 100 points, the score was positively associated with the patient's QoL.

#### 2.3.4. Complication Rate

The incidence of complications, mainly including gastrointestinal discomfort, pulmonary infection, and poststroke dementia, was calculated and compared between groups.

#### 2.3.5. Overall Response Rate (ORR)

The ORR, which was assessed with the criteria described below, was compared between the two groups: *markedly effective*: the improvement rate of neurological function was over 46% and the activities of daily living (ADL) were basically restored; *effective*: the improvement rate of the neurological function was 18%-45%, with some certain recovery of ADL; *ineffective*: the improvement rate of neurological function was less than 18%, with barely recovered ADL. ORR = markedly effective rate + effective rate.

#### 2.3.6. Nursing Satisfaction

The nursing satisfaction was also observed and compared. The nursing satisfaction questionnaire (score range: 0-100) made by our hospital was used to test the patients' satisfaction, so as to compare the nursing satisfaction score between groups. The test content and evaluation criteria were designed by ourselves, and satisfied, basically satisfied, and dissatisfied corresponded to a score of 100-85, 60-84, and below 60, respectively. Satisfaction = (satisfied cases + basically satisfied cases)/total cases∗100%.

### 2.4. Statistical Methods

SPSS v19.0 (Asia Analytics Formerly SPSS China) was used for comprehensive data analysis. *χ*^2^ test was used for the comparison of categorical data. ($\bar{\chi }\pm s$) was used to indicate the quantitative data, and *t* test was used for analysis. When *P* < 0.05, the difference was significant.

## 3. Results

### 3.1. General Information

Significant differences were absent in a series of general data such as sex, age, and average income between Groups A and B (*P* > 0.05) (Table 1).

### 3.2. Neurological Function

**Figure 1:**
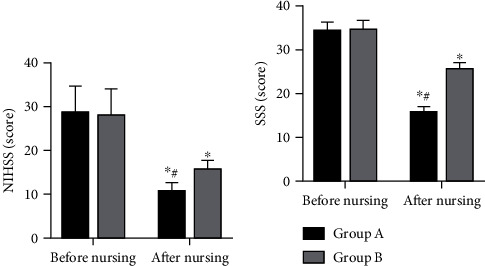
Neurological function of patients in two groups. (a) National Institutes of Health Stroke Scale (NIHSS) scores of the two groups before and after nursing. (b) Scandinavian Stroke Scale (SSS) scores of the two groups before and after nursing. Note: ^∗^*P* < 0.05 vs. after nursing; ^#^*P* < 0.05 vs. Group B.

The NIHSS score of both groups decreased one month after intervention, and the score was lower in Group A than in Group B (*P* < 0.05). One month after nursing, the SSS score decreased in both groups and was lower in Group A (*P* < 0.05) (Figure 1).

### 3.3. MH

**Figure 2:**
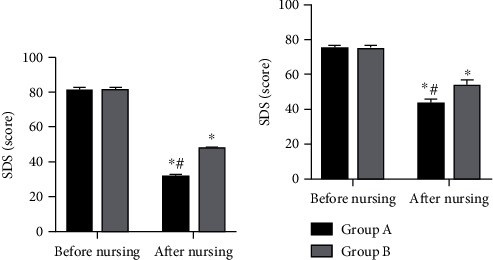
Mental health of patients in two groups: (a) Self-rating Anxiety Scale (SAS) scores of the two groups before and after nursing. (b) Self-Rating Depression Scale (SDS) scores of the two groups before and after nursing. Note: ^∗^*P* < 0.05 vs. after nursing; ^#^*P* < 0.05 vs. Group B.

SAS and SDS scores of both cohorts of patients were investigated, and it was found that there were significant changes in these scores in the two groups after nursing, with evidently lower SAS and SDS scores in Group A compared with Group B (*P* < 0.05) (Figure 2).

### 3.4. QoL

**Figure 3:**
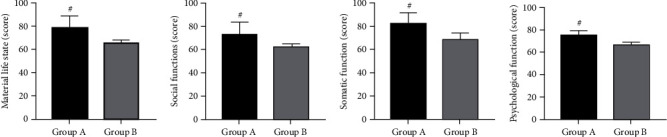
Generic Quality Of Life Inventory-74 (GQOLI-74) scores of the two groups. (a) Material life state scores of the two groups before and after nursing. (b) Social function scores of the two groups before and after nursing. (c) Somatic function scores of the two groups before and after nursing. (d) Psychological function scores of the two groups before and after nursing. Note: ^#^*P* < 0.05 vs. Group B.

After comparing the GQOLI-74 score after nursing, we found that the score of Group A was significantly higher than that of Group B (*P* < 0.05) (Figure 3).

### 3.5. Complication Rate

An evidently lower complication rate was determined in Group A compared with Group B (*P* < 0.05) (Table 2).

### 3.6. ORR

The comparison of treatment efficacy identified a higher ORR in Group A compared with Group B (*P* < 0.05) (Table 3).

### 3.7. Treatment Satisfaction

After investigation, it was found that patients in Group A were more satisfied with the care they received as compared to those in Group B (*P* <0.05) (Table 4).

## 4. Discussion

Acute stroke is a disease with a high fatality rate worldwide [22]. The treatment methods, such as acute thrombolytic therapy [23], are effective in the treatment of such patients. In addition to treatment, posttreatment patient care is also extremely important. In this section, we will combine the results of this study to discuss the influence of EBN on patients with acute stroke.

Our research results determined better neurological function in Group A intervened by EBN compared with Group B treated by routine care, similar to the research results of Ma [24]. And likewise, Dai et al. [25] showed that EBN applied to ICU patients can effectively improve their neurological function and nursing satisfaction. Following a stroke, brain nerve cells will atrophy due to the destructive effect of ischemia, among which glial cell astrocytes are more seriously damaged [26]. If such cells are damaged, the ionic and osmotic homeostasis will not be maintained, the metabolism of major neurotransmitters will be disordered, and the extracellular space will be destroyed, which will intensify inflammatory and oxidative reactions, leading to injured neurovascular function and an intact blood-brain barrier [27]. And when the nervous system is damaged, the patient's body will be affected from limbs to intestines and stomach [28, 29]. Damage to the brain is also the culprit of patients' cognitive decline, which may eventually lead to dementia [30]. In this study, Group A had better neurological recovery and therefore better brain function recovery than Group B. This is due to the fact that in the implementation process of EBN, the medical staff developed targeted treatment plans for patients, carried out more comprehensive care for patients during the whole process, and actively took measures to promote the recovery of patients in all aspects after treatment. Besides, the medical staff developed a further tacit understanding with patients via frequent communication, making patients more cooperative in the treatment process. As a result, patients in Group A have favorable treatment outcomes and better neurological recovery.

Group A was also significantly superior to Group B in terms of related scores of adverse PSY such as anxiety and depression, which indicates that EBN is more helpful to alleviate anxiety and depression than routine care. It can be found from various diseases that negative emotions will adversely affect patient recovery and aggravate the patient's condition. In stroke, it will not only induce the occurrence of stroke and destroy the function of the nervous system but also worsen the condition if the bad mood worsens after a stroke, making the condition enter a vicious circle [31]. Due to the unification of routine nursing procedures, it is inevitable that there will be corresponding deficiencies in emotion and health education management of some patients. Therefore, patients will develop anxiety about the curative effect and condition, entering the vicious circle mentioned above. While EBN is more comprehensive than conventional therapy in patients' emotional care, under such intervention, patients can have a better grasp of their own conditions, which enables patients to better manage and alleviate adverse emotions when they are anxious about their conditions [32]. As a result, patients feel more confident about their recovery and are actively involved in treatment, which naturally reduces anxiety and depression. Furthermore, the QoL of patients in Group A was significantly improved compared with Group B due to a series of posttreatment limb recovery exercises and previous good treatment results. Thus, patients recover faster, with fewer complications and higher satisfaction. Yang et al. [33] demonstrated that EBN in acute stroke patients can effectively comfort their psychological and spiritual levels, thus reducing their anxiety, depression, and other negative emotions, which is consistent with our research results. In the report of Zhang et al. [34], the application of EBN in patients with lung cancer radiotherapy and chemotherapy can not only significantly improve their psychological state but also enhance their QoL, similar to the results of our study. And previous studies have shown that the complication rate of patients with rotator cuff injury after EBN intervention is relatively lower [35], which also corroborates our findings.

The innovation of this study lies in the comprehensive analysis of the intervention effect of EBN from the aspects of neurological function, MH, QoL, complication rate, ORR, and treatment satisfaction, which confirms its effectiveness and safety in patients with acute PSD, providing a new choice for the management of such patients. But this research also shows room for improvement. This experiment only compares the advantages and disadvantages of two different nursing methods, ignoring the level of medical staff. Besides, some corresponding indicators of patients could not be studied due to the limitation of objective conditions. In the future research, we will address these defects and constantly improve the nursing methods, so as to optimize the treatment as well as nursing process.

## 5. Conclusion

In summary, EBN can better improve the PSY of patients with acute PSD, restore their neurological function, and effectively improve their QoL, which is worth promoting clinically.

## Figures and Tables

**Table 1:** General data of two groups of patients.

Categories	Group A (*n* = 100)	Group B (*n* = 50)	*t*/*χ*^2^	*P*
Gender			0.51	0.475
Male	60 (60.00)	33 (66.00)		
Female	40 (40.00)	17 (34.00)		
Age (years)	60.85 ± 6.21	59.56 ± 5.79	1.23	0.222
Average monthly income (yuan)	3355.12 ± 28.77	3359.40 ± 20.02	0.94	0.347
Place of residence			0.22	0.636
Urban	62 (62.00)	29 (58.00)		
Rural	38 (38.00)	21 (42.00)		
Marital status			0.97	0.324
Married	81 (81.00)	37 (74.00)		
Widowed/unmarried	19 (19.00)	13 (26.00)		
Drinking			0.41	0.523
Yes	73 (73.00)	34 (68.00)		
No	27 (27.00)	16 (32.00)		
Smoking				
Yes	75 (75.00)	36 (72.00)	0.15	0.693
No	25 (25.00)	14 (28.00)		

**Table 2:** Incidence of complications in the two groups.

Categories	Group A (*n* = 100)	Group B (*n* = 50)	*χ* ^2^	*P*
Gastrointestinal discomfort	3 (3.00)	5 (10.00)	—	—
Pulmonary infection	1 (1.00)	4 (8.00)	—	—
Poststroke dementia	0 (0.00)	2 (4.00)	—	—
Complication rate (%)	4 (4.00)	11 (22.00)	12.00	<0.001

**Table 3:** Total effective rate of two groups of patients.

Categories	Group A (*n* = 100)	Group B (*n* = 50)	*χ* ^2^	*P*
Markedly effective	70 (70.00)	23 (46.00)	—	—
Effective	26 (26.00)	15 (30.00)	—	—
Ineffective	4 (4.00)	12 (24.00)	—	—
Total effective rate (%)	96 (96.00)	38 (76.00)	13.99	<0.001

**Table 4:** Satisfaction of two groups of patients.

Categories	Group A (*n* = 100)	Group B (*n* = 50)	*χ* ^2^	*P*
Satisfied	72 (72.00)	19 (38.00)	—	—
Basically satisfied	21 (21.00)	16 (32.00)	—	—
Dissatisfied	7 (7.00)	15 (30.00)	—	—
Treatment satisfaction (%)	93 (93.00)	35 (70.00)	14.09	<0.001

## Data Availability

The labeled dataset used to support the findings of this study are available from the corresponding author upon request.
